# Musculoskeletal ultrasound versus magnetic resonance in supporting clinical management of juvenile idiopathic arthritis

**DOI:** 10.1186/1546-0096-12-S1-O15

**Published:** 2014-09-17

**Authors:** Stefano Lanni, Erica Ricci, Angela Pistorio, Francesca Magnaguagno, Angelo Ravelli, Stefania Viola, Marta Dellepiane, Alberto Martini, Clara Malattia

**Affiliations:** 1 IRCCS Istituto Giannina Gaslini, Italy; 2 Universita' di Genova, Genova, Italy

## Introduction

Over the last decade the use of musculoskeletal ultrasound (MSUS) for the assessment of juvenile idiopathic arthritis (JIA) has increased considerably. However, little is known about the potential of MSUS in supporting the clinical management of JIA, when compared to magnetic resonance (MR).

## Objectives

To evaluate whether MSUS is able to equate MR in providing the information desired by the clinician in practice situations of patients with JIA.

## Methods

Fifty-nine consecutive children with JIA who performed a joint MR were scanned at the same day also with MSUS. Overall, 23 patients were assessed by imaging in the wrist, 13 in the hips, 12 in the ankle, 4 in the temporomandibular joints (TMJs), 5 in the knee, and 2 in the shoulder. The physician was requested to specify the clinical indication for which the MR was prescribed. MR and MSUS pathological findings were defined according to OMERACT definitions. Concordance between MR and MSUS results was tested using Cohen’s kappa coefficient.

## Results

MR was requested for: 1) confirming disease remission, 2) assessing disease activity, 3) evaluating presence of structural damage. Twenty-five patients were in clinical remission; both MR and MSUS confirmed remission in 10/25 (40%) patients, whereas both imaging modalities revealed active disease in other 10/25 (40%) patients. In the remaining 5 (20%) patients, remission was confirmed only by MR or MSUS in 1 and 4 patients, respectively. Concordance between MR and MSUS for evaluating disease remission was good (k=0.61). In the 34 patients with clinically active JIA, both imaging modalities confirmed active disease in 23/34 (68%) patients. Ten/34 (29%) patients had no signs of active disease on MSUS, but only 4 (40%) of them showed inactivity on MR. Concordance between MR and MSUS for evaluating disease activity was moderate (k=0.42). MR and MSUS agreed on the presence of structural damage in 7 out of 9 patients whose MR was requested also for evaluating joint damage. In 1 patient damage was revealed only by MSUS, and in the remaining patient no damage was depicted by both imaging modalities. Irrespective of the clinical question, the percentages of agreement between MR and MSUS for each joint were: 100% for knee and shoulder, 85% for hips, 83% for ankle, 74% for wrist, and 50% for TMJs.

## Conclusion

MSUS is able to equate MR in assessing patients in clinical remission. The two imaging modalities show a moderate concordance in evaluating disease activity. Unexpectedly, MSUS seems as useful as MR in demonstrating structural damage. MR is more suitable than MSUS for the assessment of TMJs and wrist.

## Disclosure of interest

None declared.

